# Thromboprophylaxis prescribing among junior doctors: the impact of educational interventions

**DOI:** 10.1186/s12913-016-1480-9

**Published:** 2016-07-15

**Authors:** Bethany J. Watt, Dean T. Williams, Lauren Lewis, Christopher J. Whitaker

**Affiliations:** Department of Vascular Surgery, Ysbyty Gwynedd, Bangor, LL57 2PW UK; School of Medical Sciences, Bangor University, Bangor, Gwynedd LL57 2DG UK; North Wales organisation for Randomised Trials in Health, Bangor University, Bangor, Gwynedd LL57 2DG UK

**Keywords:** Thromboprophylaxis, Thromboembolism, Surgery, Compliance, Prescription, Teaching, Pro-forma

## Abstract

**Background:**

Venous thromboembolism (VTE) prophylaxis in an important aspect of the care of hospitalised patients, for which the National Institute for Health and Care Excellence (NICE) has issued guidance. Guidance compliance continues to be a concern. Junior doctors are the main group responsible for prescribing thromboprophylaxis. We aimed to compare local pharmacological thromboprophylaxis prescribing against NICE guidelines in a surgical department at a district general hospital, and determine whether interventions aimed at improving compliance were effective.

**Methods:**

Over four months, a two cycle audit of prescribing patterns for VTE prophylaxis was performed using data collected at four intervals: 1. Baseline 2. Following pro-forma introduction and feedback 3. A second baseline data collection. 4. Following VTE prophylaxis teaching.

**Results:**

A total of 394 admissions were included. Correct identification and prescribing for at-risk patients ranged between 76 and 93 %, whilst risk assessment documentation and explanation to patients occurred in fewer than 50 and 66 % respectively. Prescribing and risk assessment improved in the first cycle (chi2 = 6.75, *p* = 0.009 and chi2 = 10.70, *p* = 0.001 respectively), a consequence of one specialty improving following additional feedback. Teaching was not associated with improvements. Overall compliance with NICE guidelines was achieved in no more than 25 % of admissions.

**Conclusions:**

Despite junior doctors generally prescribing VTE thromboprophylaxis appropriately, overall compliance with guidelines remained poor regardless of educational interventions. Verbal feedback was the only intervention associated with modest improvements. A pressurised work environment may limit the impact of educational interventions. Guidance simplification or devolving responsibility to other members of staff may improve compliance.

**Electronic supplementary material:**

The online version of this article (doi:10.1186/s12913-016-1480-9) contains supplementary material, which is available to authorized users.

## Background

Venous thromboembolism (VTE) is a major cause of morbidity and mortality, accounting for as many as 25,000 deaths amongst hospitalised patients in the UK each year, [1]. The term refers to occlusion within the venous system by a blood clot, which may or may not become dislodged from its site of origin, and encompasses both deep vein thrombosis and its most dangerous complication, pulmonary embolism (PE). Without prophylactic treatment, VTE is estimated to occur in 29 % of all surgical patients, and in as many as 60 % of those undergoing elective orthopaedic surgery, [2]. However, with the effective use of pharmacological thromboprophylaxis, these figures are significantly reduced, [3].

The National Institute for Health and Care Excellence (NICE) has developed guidelines outlining the best practice for reducing the risk of VTE in hospitalised patients, which include the prescribing of pharmacological thromboprophylaxis in the form of anticoagulation for at-risk patients, [2]. Campaigns such as the ‘1000 lives plus’, used by our unit, have sought to raise awareness of this important aspect of healthcare, [4]. Despite the large body of evidence supporting the use of VTE prophylaxis, literature still suggests low rates of adherence amongst hospital staff both in the UK and worldwide, [5]. At this surgical unit, previous small audits and anecdotal evidence suggested that thromboprophylaxis prescribing was also sub-optimal. There are many published studies on the impact of initiatives to improve VTE prophylaxis compliance that include various types of feedback, pro-forma, order sets, focussed teaching and information provision [6–9]. Interventions are generally reported to have positive impacts on performance, but generally more inclusive, active initiatives appear to have greater impact than the more passive provision of information and attendance on teaching sessions. However, studies in this area employ very different methodologies, including retrospective and prolonged data collection and often small numbers of participants, and are vulnerable to confounding variables, [10]. The value of continuing medical education strategies aimed at improving clinical performance has been debated for many years, but it is recognised that the engagement of those targeted in the educational activity is a key determinant of success, [11, 12]. Educational interventions for new prescribers, particularly relevant to the junior doctors responsible for the majority of prescribing in VTE prophylaxis, generally prove positive, but although audit and feedback have been demonstrated to positively influence practice, it is unclear as to which interventions are most effective [13, 14].

## Aims & objectives

### Aims

This audit aimed to determine adherence to NICE and local guidelines with regard to the pharmacological VTE prophylaxis practice among junior doctors working in three units of a surgical department at a district general hospital and prospectively determine whether two interventions, a pro-forma decision support tool and teaching intervention intended to improving clinical performance, were associated with improved guidance adherence.

### Objectives

- To assess adherence to NICE and local guidelines with regard to pharmacological VTE prophylaxis within the surgical department of a district general hospital.
- To identify areas for improvement in the prescription of pharmacological VTE prophylaxis.
- To implement strategies to improve adherence to NICE and local guidelines.
- To measure compliance following the introduction of those strategies.

## Methods

A form was designed to capture data on pharmacological VTE prophylaxis based on NICE guidelines (Additional file 1).

**Fig. 1:**
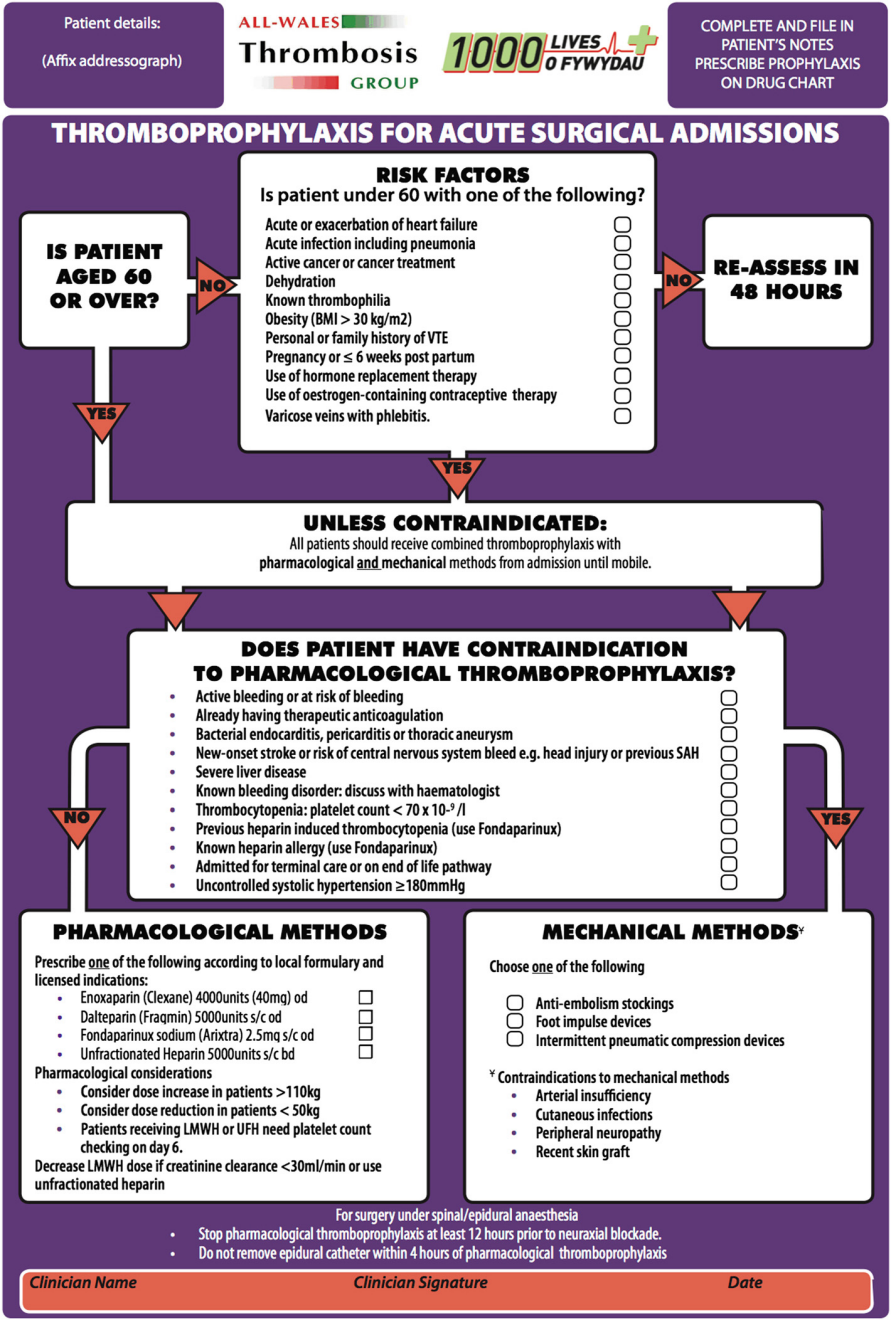
The '1000 Lives +' pro-forma employed for acute surgical admissions

Data was retrieved at four key points over two audit cycles: 1. Baseline current practice, 2. Following introduction on the wards of an ‘Acute Surgery Risk Assessment’ pro-forma tool, provided by the All-Wales ‘1000 lives +’ campaign (Fig. 1), 3. A second new baseline assessment (that included new junior doctors), and 4. Audit cycle completion approximately two weeks later following a mandatory teaching session on NICE and local guidelines for VTE prophylaxis.

The ‘1000 lives plus’ pro-forma, designed for inclusion in patient notes, provides a flow chart for assessing VTE risk factors and contraindications to pharmacological thromboprophylaxis in-line with NICE guidelines, and allows for documentation of risk assessment, [4]. Copies of the tool were displayed above the ward trolleys containing patients’ medical notes. Junior medical staff on each ward were informed verbally of the pro-forma introduction and availability.

To gain a more accurate picture of all junior staff VTE prophylaxis activity on the surgical unit, data was retrieved from treatment charts and notes of inpatients on the vascular, urology and general surgical wards on two occasions 14 days apart on each of the four rounds of the audit. The two unannounced snapshots of activity ensured that the vast majority of junior doctors, mainly foundation years one and two, but also core trainees and clinical fellows, were included in the study. All prescription charts were completed by junior doctors. Details on patients receiving VTE information were retrieved from medical notes, nursing notes, prescription charts, verbal communication with staff and by asking patients themselves. Where there was no evidence, either in the form of documentation of a discussion with the patient or patient recollection, information was presumed not to have been given.

The findings of the first completed audit cycle, employing a pro-forma, were presented to Foundation Year 1 junior doctors as part of their mandatory teaching programme. The teaching session focussed on areas targeted for improvement based on results from the first audit cycle. The second baseline assessment and the educational intervention in the form of this teaching formed the basis of the second audit cycle. The second baseline VTE compliance assessment was performed prior to the teaching session to identify any variance in prescribing practice or documentation that might have occurred as a result of new foundation doctors joining the surgical teams. The great majority of the junior foundation doctors had previously worked in medical posts at the same institution as part of a twelve month placement rotating through medicine and surgery.

One further educational intervention in the form of verbal feedback was given to senior medical staff in one specialty following the baseline data collection in the first audit cycle. This was an additional intervention triggered by clinical concern regarding poor compliance with thromboprophylaxis guidelines and occurred at the same time as the introduction of the pro-forma.

Data was analysed using the statistical software programme SPSS 20. Frequencies and percentages were used to draw comparisons between subgroups of audited admissions and chi2 was employed for statistical analysis for non-parametric data, significance was presumed at *p* ≤ 0.05. Where information could not be retrieved, the data was entered as unknown and not included in subsequent analyses.

This study did not require ethical approval or patient consent; the audit was registered locally with the audit department as a study of clinical service provision.

## Results

A total of 394 patient admissions were included in the study, the majority of which were emergencies (85 %) equally distributed across all three wards. These comprised 124 cases in the initial assessment, 77 following the introduction of the pro-forma, 98 s baseline assessments after the changeover of doctors, and 95 subsequent to the teaching session.

Table 1 (end of manuscript) presents the combined data from all three surgical specialties for each of the two audit cycles. As demonstrated, 80.6 % of patients were prescribed the anti-coagulant low molecular weight heparin enoxaparin in the first baseline analysis, compared with 92.9 % following the introduction of the pro-forma. The second baseline analysis, following the rotation of Foundation trainees, demonstrated 75.7 % of admissions received enoxaparin vs. 78.5 % following the teaching session. Statistical analysis demonstrated that the improvement in appropriate prescribing (when indications were present and contra-indications absent) following introduction of the pro-forma were significant, (chi2 = 8.79, *p* = 0.032). Teaching was not associated with demonstrable changes in appropriate prescribing performance, (chi2 = 0.01, *p* = 0.768). There was no significant difference in the appropriate prescribing compliance between the two different cohorts of junior doctors at baseline (chi2 = 3.16, *p* = 0.075). Table 1 also illustrates that the correct dose of anti-coagulant was prescribed in over 93 % of patients prescribed thromboprophylaxis at all data collection points. Administration of anticoagulant once prescribed occurred in over 94 % of patients and was given within 24 h in over 90 % of all patients except in the pre pro-forma group where it was 83 %. Prescribing of anti-coagulant when there was a contra-indication occurred at all data collection points, (8 to 30 % of patients) and was not associated with improvements following interventions (chi2 = 3.21, *p* = 0.36).

**Table 1:** Adherence to NICE and local VTE pharmacological prophylaxis guidelines (all specialties combined)

Parameter measured	(N) Number of patients included			
	Pre-pro-forma (*N* = 124)	Post- pro-forma (*N* = 77)	Post- changeover of doctors (*N* = 98)	Post-teaching (*N* = 95)
Indication(s) for pharmacological prophylaxis present, % (number)	y = 94.4 % (117) n = 5.6 %(7)	y = 88.3 % (68) n = 10.4 % (8)	y = 92.9 % (91) n = 6.1 % (6)	y = 93.7 % (89) n = 6.3 % (6)
Contraindication(s) to pharmacological prophylaxis present, % (number)	y = 10.5 % (13) n = 87.9 % (109) u/n = 1.6 % (2)	y = 19.5 % (15) n = 80.5 %(62)	y = 13.3 % (13) n = 81.8 % (81) u/n = 4.9 % (4)	y = 26.3 % (25) n = 73.7 % (70)
**Documentation of risk assessment, % (number)**	**y = 21.8 % (27)** **n = 75.8 % (94)** **u/n = 2.4 % (3)**	**y = 42.9 % (33)** **n = 53.2 % (41)** **u/n = 3.9 % (3)**	**y = 42.9 % (42)** **n = 49.0 % (48)** **u/n = 8.1 % (8)**	**y = 35.8 % (34)** **n = 61.1 % (58)** **u/n = 3.1 % (3)**
**Information regarding prophylaxis given to patient, % (number)**	**y = 46 % (57)** **n = 35.5 % (44)** **u/n = 18.5 % (23)**	**y = 41.6 % (32)** **n = 40.3 % (31)** **u/n = 18.2 % (14)**	**y = 32.7 % (32)** **n = 34.7 % (34)** **u/n = 32.7 % (32)**	**y = 45.3 % (43)** **n = 24.2 % (23)** **u/n = 30.5 % (29)**
Patients prescribed pharmacological prophylaxis, % (number)	y = 72.6 % (90) n = 25.8 % (32) u/n = 1.6 % (2)	y = 74 % (57) n = 23.4 % (18) u/n = 2.6 % (2)	y = 61.2 % (60) n = 35.7 % (35) u/n = 3.1 % (3)	y = 62.1 % (59) n = 36.8 % (35) u/n = 1.1 % (1)
**Patients prescribed pharmacological prophylaxis, where indication(s) present and contraindication(s) absent, % (number)**	**Y = 80.6 % (83)** **n = 18.4 % (19)** **u/n = 1 %(1)**	**y = 92.9 % (52)** **n = 3.6 % (2)** **u/n = 3.6 %(2)**	**y = 75.7 % (56)** **n = 21.6 % (16)** **u/n = 2.7 % (2)**	**y = 78.5 % (51)** **n = 20 % (13)** **u/n = 1.5 % (1)**
Patients prescribed pharmacological prophylaxis where contraindication(s) present, % (number)	y = 30.8 % (4) n = 69.2 % (9)	y = 20 % (3) n = 80 % (12)	y = 7.7 % (1) n = 92.3 % (12)	y = 32 % (8) n = 68 % (17)
Adherence to prescribed pharmacological prophylaxis, (signed as given) % (number)	y = 94.4 % (85) n = 1.1 % (1) u/n = 4.4 % (4)	y = 94.3 % (50) n = 5.7 % (3) u/n = 7 % (4)	y = 95 % (57) n = 2.0 % (1) u/n = 3.3 % (2)	y = 98.3 % (58) n = 1.7 % (1)
Correct dose of pharmacological prophylaxis prescribed, % (number)	y = 93.3 % (70) n = 6.7 % (5) u/n = 16.7 % (15)	y = 93 % (53) n = 7 % (4)	y = 93.3 % (56) n = 6.7 % (4)	y = 96.6 % (57) n = 3.4 % (2)
How soon after admission pharmacological prophylaxis prescribed, % (number)	<24 h = 82.2 % (74) >24 h = 13.3 % (15) u/n = 1.1 % (1)	<24 h = 91.2 % (52) >24 h = 8.8 % (5)	<24 h = 96.7 % (58) >24 h = 3.3 % (2)	<24 h = 91.5 % (54) >24 h = 6.7 % (4) u/n = 1.7(1)
Type of pharmacological prophylaxis prescribed, % (number)	Enoxaparin = 99.2 % (89) Unfractionated heparin = 0.8 (1)	Enoxaparin= 100 % (57)	Enoxaparin = 98.3 % (59) Unfractionated heparin = 1.7(1)	Enoxaparin = 100 % (59)

This table shows prescribing-related data for all patients prior to and following interventions amongst all specialties combined. The rows relate to the prescribing of pharmacological thromboprophylaxis, including indication, dose and timing and whether anti-coagulation was indicated or contra-indicated. The three rows highlighted in bold text relate to: 1. The documentation of an assessment of risk, 2. The provision of information to patients and 3. Whether a patient was appropriately prescribed anti-coagulation when indicated and not contra-indicated. These highlighted rows represent key measures of compliance related to NICE guidelines. The third highlighted row relates to the proportion of appropriate pharmacological thromboprophylaxis prescribing where there is an indication and an absence of known contraindications and is calculated from the prescribing data captured in the rows above

*Key*: *y* yes, *n* no, *u/n* unknown

Analysis of the appropriate prescribing data per specialty (Table 2), revealed that compliance within the Urology Department was reduced at 68.6 % vs. 78.4 % and 91.4 % for General and Vascular surgical departments respectively (chi2 = 10.0386, *p* = 0.007).

**Table 2:** Proportion of compliant VTE prophylactic pharmacological (prescribing per specialty all data combined)

Parameter Measured	(N) Number of patients included		
	General Surgery (*N* = 177)	Urology (*N* = 51)	Vascular Surgery (*N* = 70))
Patients prescribed pharmacological VTE prophylaxis, where indication(s) present *and* contraindication(s) absent, % (number)	y = 78.4 % (134) n = 21.6 % (37) u/n = 3.4 % (6)	y = 68.6 % (35) n = 31.4 % (16)	y = 91.4 % (64) n = 8.6 % (6)

This table shows prescribing-related data for patients admitted under the three main surgical specialties across all rounds of data collection, and relates to the proportion of appropriate pharmacological thromboprophylaxis prescribing where there is an indication and an absence of known contraindications

*Key*: *y* yes, *n* no, *u/n* unknown

Further analysis of the Urology data is presented in Table 3 (end of manuscript), and demonstrates that appropriate prescribing compliance at the first snapshot data collection was only 35.7 %, rising to 100 % following introduction of pro-forma and provision of feedback, and remaining at 81 % and 82 % in the second audit cycle (chi2 = 9.7093, *p* = 0.002).

**Table 3:** Adherence to NICE and local VTE pharmacological prophylaxis guidelines (urology department)

Parameter Measured	(N) Number of patients included			
	Pre-proforma (*N* = 25)	Post- proforma (*N* = 12)	Post- changeover of doctors (*N* = 28)	Post-teaching (*N* = 19)
Indication(s) for pharmacological VTE prophylaxis present, % (number)	y = 92.0(23) n = 8.0(2)	y = 66.7(8) n = 33.3(4)	y = 96.4(27) n = 3.6(1)	y = 100(19)
Contraindication(s) for pharmacological VTE prophylaxis present, % (number)	y = 32.0(8) n = 64.0(16) u/n = 4.0(1)	y = 41.7(5) n = 58.3(7)	y = 17.9(5) n = 78.6(22) u/n = 3.6(1)	y = 42.1(8) n = 57.9(11)
**Documentation of risk assessment, % (number)**	**y = 8.0 %(2)** **n = 88.0 %(22)** **u/n = 4.0 %(1)**	**y = 25.0 %(3)** **n = 66.7 %(8)** **u/n = 8.3 %(1)**	**y = 32.1 %(9)** **n = 57.1 %(16)** **u/n = 10.7 %(3)**	**y = 31.6 %(6)** **n = 68.4 %(13)**
**Information regarding VTE prophylaxis given to patient, % (number)**	**y = 28.0 %(7)** **n = 56.0 %(14)** **u/n = 16.0 %(4)**	**y = 50.0 %(6)** **n = 41.7 %(5)** **u/n = 8.3 %(1)**	**y = 35.7 %(10)** **n = 28.6 %(8)** **u/n = 35.7 %(10)**	**y = 57.9 %(11)** **n = 21.1 %(4)** **u/n = 21.1 %(4)**
Total patients prescribed pharmacological VTE prophylaxis, % (number)	Y = 32(8) N = 68(17)	y = 33.3(4) n = 58.3(7) u/n = 8.3(1)	y = 60.7(17) n = 35.7(10) u/n = 3.6(1)	y = 63.2(12) n = 36.8(7)
**Patients prescribed pharmacological VTE prophylaxis, where indication(s) present** ***and*** **contraindication(s) absent, % (number)**	**Y = 35.7 %(5)** **N = 64.3 %(9)** **(total = 14)**	**y = 100 %(4)** **u/n = 20 %(1)** **(total 5)**	**y = 81 %(17)** **n = 19 %(4)** **(total 21)**	**y = 81.8 %(9)** **n = 18.2 %(2)** **(total 11)**
Patients prescribed pharmacological prophylaxis where contraindication(s) present, % (number)	Y = 25.0(2) N = 75(6) (Total =8)	n = 100(5) (total 5)	n = 100(5) (total 5)	y = 37.5(3) n = 62.5(5) (total 8)
Adherence to prescribed pharmacological VTE prophylaxis, % (number)	y = 32(8) u/n = 68(17)	y = 25.0(3) n = 8.3(1) u/n and n/a = 66.6(8)	y = 57.1(16) n = 3.6(1) u/n = 39.3(11)	y = 63.2(12) n/a = 36.8(7)
Correct dose of pharmacological VTE prophylaxis prescribed, % (number)	Y = 20(5) N = 12(3) n/a = 68(17)	y = 33.3(4) n = 66.7(8)	y = 60.7(17) n/a = 39.3(11)	y = 63.2(12) n = 36.8(7)
How soon after admission pharmacological VTE prophylaxis prescribed, % (number)	<24 h = 16(4) >24 h = 16(4) n/a = 68(17)	<24 h = 33.3(4) u/n and n/a = 66.6(8)	<24 h = 57.1(16) >24 h = 3.6(1) n/a = 39.3(28)	<24 h = 47.4(9) >24 h = 15.8(3) n/a = 36.8(7)
Type of pharmacological VTE prophlyaxis prescribed, % (number)	Enoxaparin = 32 (8) Unfract.Hep u/n = 68(17)	Enoxaparin = 33.3 (4) None/na = 66.7(8)	Enoxaparin =60.7(17) Unfract Hep 3.6 (1) n/a = 35.7(10)	Enoxaparin = 63.2(12) None/na = 36.8(7)

This table shows prescribing-related data for patients prior to and following interventions within the urology department. The rows relate to the prescribing of pharmacological thromboprophylaxis, including indication, dose and timing and whether anti-coagulation was indicated or contra-indicated. The three rows highlighted in bold text relate to: 1. The documentation of an assessment of risk, 2. The provision of information to patients and 3. Whether a patient was appropriately prescribed anti-coagulation when indicated and not contra-indicated. These highlighted rows represent key measures of compliance related to NICE guidelines. The third highlighted row relates to the proportion of appropriate pharmacological thromboprophylaxis prescribing where there is an indication and an absence of known contraindications and is calculated from the prescribing data captured in the rows above

*Key*: *y* yes, *n* no, *u/n* unknown

Our data demonstrates that for documentation of risk, compliance was never greater than 50 %. However, baseline data in the first cycle demonstrated documentation in 21.8 % of admission notes, improving to 42.9 % following pro-forma introduction (chi2 = 9.88, *p* = 0.002). Again, this improvement reflected changes in compliance in Urology, improving from 8 to 25 % following pro-forma introduction and feedback, and maintaining at approximately 32 % at subsequent data collection points (chi2 = 5.2588, *p* = 0.02). Teaching was not associated with changes in risk documentation, 42.9 % pre- vs.35.8 % post-teaching (chi2 = 1.86, *p* = 0.173).

Information provided to patients was relatively constant throughout the audit cycles. Although there were up to 1/3 of unknowns in this data, information was given in approximately 50 to 66 % of admissions, with no improvement associated with interventions (chi2 = 4.4023, *p* = 0.22).

Overall, compliance with NICE and local guidelines, based on the individual data contained in the six rows in Table 1 relating to appropriate prescribing, risk assessment, patient information, dose, administration and timing for each patient, was achieved in only 17/124 (13.7 %), 19/77 (24.7 %), 16/98 (16.3 %) and 19/95 (20 %) of patient admissions across the four points of the study.

## Discussion

This detailed audit of VTE pharmacological prophylaxis in a district general hospital has demonstrated that compliance with national guidelines was variable and overall poor. The audit further demonstrated that some areas of practice were consistently more prone to poor compliance. Documentation of risk assessment and provision of patient information was persistently poorly performed. Variations in the baseline appropriate prescribing (where indications were present and contra-indications absent) between the two audit cycles were not statistically significant. Appropriate prescribing of anticoagulation occurred in 76 to 93 % of patients. The introduction of pro-forma was associated with improvements when data from all three specialties were combined (Table 1). No changes in VTE thromboprophylaxis prescribing performance were demonstrated following teaching.

However, further analysis of the performance of appropriate prescribing per specialty (Table 2), demonstrates that Urology performed relatively poorly. Analysis of baseline appropriate prescribing data contained in Table 3 for Urology in the first cycle demonstrated a compliance of 36 % compared to 80 % for all specialties combined (illustrated in Table 1). Improvements in prescribing on the Urology ward were maintained at between 80 % and 100 % following the introduction of a pro-forma. However, Urology was the only specialty to have an additional educational intervention in the form of verbal feedback. This improvement achieved levels of compliance comparable with the combined results for all specialties in the first audit cycle and was related to the overall improvements demonstrated. The initially better performing specialties of General and Vascular Surgery were unaffected by pro-forma or teaching.

The additional feedback discussions with that Urology senior staff regarding VTE prophylaxis were triggered by the results of the initial snap shot of practice that demonstrated that the majority of patients did not receive enoxaparin (Table 3). It is worth noting that when compared to other surgical specialties, Urology has a higher proportion of patients with contra-indications to pharmacological prophylaxis (Tables 1 and 3), largely related to haematuria as a presenting condition. Despite this improvement, 20 % of patients across all specialties remained at elevated risk of VTE due to failure to prescribe enoxaparin.

The particularly poor compliance in documentation of VTE risk assessment within the Department of Urology also improved following feedback to senior staff and the introduction of the pro-forma. In the better performing specialties, both documentation and provision of information were not associated with changes following the introduction of pro-forma or teaching. It is likely that the junior doctors working in Urology were as familiar with VTE thromboprophylaxis as those working in other specialties. Whilst it is possible that the pro-forma enhanced compliance when performance was particularly poor, based on the discussions with senior staff in Urology, it is likely that the inclusion of verbal feedback was responsible for the changes identified in this one specialty.

Our findings suggest that feedback may be as effective as multiple other educational approaches in improve prescribing practice, particularly when approached in a constructive manner with colleagues who are receptive and willing to improve practice [15, 16]. Whilst it is the responsibility of all members of the surgical team to consider pharmacological thromboprophylaxis, it is of particular relevance to those doctors set with the task of ‘clerking in’ the new patient. Initial assessment and drug prescribing is ordinarily done by a range of more junior doctors, in particular Foundation Year 1 and 2 doctors, and Core Surgical Trainees. As mentioned in the background text, educational interventions for new prescribers is of particular importance, [14]. The practice of these doctors may be influenced by the senior clinicians within the team who can include feedback as part of patient centred learning activities, [17]. The Urology team were identified as being initially particularly poorly compliant and were the only specialty to be given verbal feedback. This one-off verbal feedback given by a colleague to a senior clinician in a poorly performing team is a method of education that may be more likely to affect change [6].

Consistent with a previous audit study on our unit, this audit suggests that a ceiling may be reached where, for this junior doctor population working in an emergency environment, there is a limit to the degree of compliance that can be achieved against current VTE guidelines, regardless of the type of interventions intended to improve performance. The efficacy of educational programmes targeted at improving performance in elective, less pressured environments with greater levels of resource, may not apply so readily to the acute environments with greater demands, regardless of individuals’ resourcefulness [18, 19]. The negative effects of busy working environments causes individuals to prioritise key and relatively simple tasks and focus less on more complex activities that involve decision making and require greater amounts of time [20, 21].

### Limitations

The study included multiple educational activities including feedback through teaching and the introduction of a pro-forma for all specialties. Verbal feedback was also included as a single intervention for one poorly performing specialty only. Although this was a detailed study, the audit concerned pharmacological prophylaxis only and did not assess other aspects of VTE prevention. The data reflects junior doctors’ practice in one district general hospital. The vast majority of junior doctors were from a single U.K. medical school, with the remaining from other U.K. medical schools. They were therefore all familiar with VTE prophylaxis guidelines. There were seven foundation year 1 doctors working within the surgical department at any one time during the year in which the audit was conducted, with two of these rotating to other surgical posts after the first four months in which the initial snapshots of data had been collected. There were also a similar number of more senior Foundation Year 2 and/or Core Surgical Trainee doctors working at the time, whose prescribing practices would also have been reflected in the audit. Although these doctors would have been exposed to the introduction of the pro-forma, they did not receive any additional educational interventions. Any variations in compliance related to changes in the junior doctors were accommodated by including a second baseline’ pre-intervention data collection in the second audit cycle. However, the doctors and patients included in the second cycle represent different populations and may have influenced the data. Middle grade and Consultant staff members were not targeted by the pro-forma and teaching interventions of this audit, but received verbal feedback in the initially poorly performing specialty. Feedback to senior staff in Urology during the first cycle is likely to have influenced the second audit cycle results.

## Conclusions

The prescribing practice for VTE prophylaxis among junior doctors on surgical wards was demonstrated to expose approximately 10 to 25 % of patients, the majority emergency admissions, to unnecessary risk*.* Compliance with all key aspects of VTE guidance was achieved in approximately 25 % of patients. This two cycle audit demonstrated that two educational interventions, pro-forma and focused teaching aimed at improving VTE pharmacological thromboprophylaxis, were not associated with changes in practice in departments initially achieving over 70 % compliance in appropriate prescribing. However, where verbal feedback was given to senior staff in addition to the introduction of the pro-forma decision support tool in one poorly performing specialty, compliance with appropriate prescribing and documentation of risk assessment were associated with improvements in practice to levels similar to the other surgical specialties. If we assume that junior doctors’ knowledge and ability to understand guidelines is sound, then this audit suggests that their performance can be influenced by senior staff, but it is the environment in which they work that has a significant impact on their ability to achieve compliance. These results raise concerns that the addition of more complex and time consuming protocols and guidelines to the doctor’s workload in the acute environment may not achieve the intended improvements in patient safety. In the context of the current junior doctor’s working environment when receiving emergency admissions, awareness and checks of adherence by other staff and simplification of guidelines may help to improve practice and reduce the risk of VTE.

## Abbreviations

NICE, National Institute for Health and Care Excellence; PE, pulmonary embolism; VTE, venous thromboembolism.

## Additional file

Additional file 1: Pro forma for performance data collection. (DOC 26 kb)
