# Supplementary material for: Thromboprophylaxis prescribing among junior doctors: the impact of educational interventions
Source: BMC Health Serv Res. 2016 Jul 15;16:267. doi: 10.1186/s12913-016-1480-9 (PMC4946149; doi:10.1186/s12913-016-1480-9)
Supplement: Additional file 1: — Pro forma for performance data collection. (DOC 26 kb) [file 12913_2016_1480_MOESM1_ESM.doc]

Figure 1 Pro forma for performance data collection

**Patient demographics**

- Age (18-30) (31-40) (41-50) (51-60) (61-70) (71-80) (81-90) (91+)
- Gender M / F
- Surgical team (General) (Vascular) (ENT) (Orthopaedic) (Other)
- Presenting complaint:

**Thromboprophylaxis**

Is there an indication for VTE prophylaxis? Y / N / DK

- Total anaesthetic + surgical time > 90minutes
- Surgery to pelvis / lower limb + total anaesthetic + surgical time > 60 minutes
- Acute surgical admission with inflammatory or intra-abdominal condition
- Expected to have significant reduction in mobility
- Any VTE risk factor present

Is there a contraindication to pharmacological VTE prophylaxis?

Y / N / DK

- Active bleeding
- Acquired bleeding disorders (e.g. acute liver failure)
- Concurrent use of anticoagulants
- LP/epidural/spinal anaesthesia within previous 4 hours or expected within next 12 hours
- Acute stroke
- Thrombocytopaenia (platelets < 75)
- Uncontrolled systolic hypertension (>230/120mmHg)
- Untreated inherent bleeding disorders

Is there documentation of assessment of patient for risk of VTE and bleeding?

Y / N / DK

Has patient been provided with information regarding VTE prophylaxis?

Y / N / DK

Is pharmacological VTE prophylaxis prescribed? Y / N / DK

If Y, what type?

- LMWH
- UFH
- other

If Y, is thromboprophylaxis being adhered to? Y / N / NA

If pharmacological thromboprophylaxis, is this prescribed at the appropriate dose?

- Y
- N (please specify)

When was pharmacological VTE prophylaxis prescribed?

Within fist 24hours of admission After first 24hours of admission
